# Perspectives on hospice and palliative care in an academic setting: An exploratory interview study with patients and relatives to inform the development of an academic inpatient hospice

**DOI:** 10.1371/journal.pone.0348513

**Published:** 2026-05-07

**Authors:** Mareike Löbberding, Heidrun Golla, Anna Wolf, Sukhvir Kaur, Steffen T. Simon, Veronika Dunkl, Julia Strupp, Raymond Voltz, Kerstin Kremeike

**Affiliations:** 1 Faculty of Medicine and University Hospital, Department of Palliative Medicine, University of Cologne, Cologne, Germany; 2 Department of Palliative Medicine, University Medical Center Göttingen, Goettingen, Germany; 3 Faculty of Medicine and University Hospital, Center for Integrated Oncology Aachen-Bonn-Cologne-Duesseldorf (CIO ABCD), Cologne, Germany; 4 Faculty of Medicine and University Hospital, University of Cologne, Center for Health Services Research, Cologne, Germany; UNBC: University of Northern British Columbia, CANADA

## Abstract

**Background:**

Although palliative and hospice care are essential for individuals with life-limiting illnesses, academic inpatient hospices, which combine care with research and education, remain rare. This study explores the experiences, perceptions, and expectations of patients and their relatives regarding palliative and hospice care with the aim of informing the development of an academic inpatient hospice.

**Methods:**

Semi-structured individual and dyadic interviews were conducted with patients and relatives who had experience with palliative and hospice care services in an academic setting in Germany. The data were analyzed using qualitative content analysis.

**Results:**

A total of 14 interviews were conducted with 17 participants (10 patients, 7 relatives). Participants reported initial uncertainty and skepticism regarding palliative and hospice care; however, direct experiences led to more positive attitudes. Key aspects valued included psychosocial support, effective symptom management, and a non-clinical atmosphere. The preservation of autonomy and dignity, meaningful personal interactions, and the active involvement of relatives were considered essential. The transition from a hospital-based palliative care unit to an inpatient hospice was often perceived as abrupt and emotionally challenging, creating a risk of losing established familiarity. Participants responded positively to the idea of an academic inpatient hospice. They highlighted the potential benefits of integrating palliative and hospice services within a unified and familiar environment, including smoother transitions and continuity of care.

**Conclusions:**

An academic inpatient hospice offers opportunities to address end-of-life care needs by strengthening existing structures and ensuring continuity and comprehensiveness of care. It can support patient-centered care, provide a platform for education and research, and promote greater public awareness and understanding of palliative and hospice care.

## Introduction

The World Health Organization (WHO) is engaged in a range of initiatives aimed at enhancing palliative and hospice care worldwide [1]. Despite these efforts, several challenges persist, including the increasing demand for such services due to an aging population and the rising incidence of chronic diseases [2,3]. Financial constraints and resource limitations also pose significant challenges to the expansion and improvement of palliative and hospice care [4].

While hospice care is conceptually understood as a specific form of palliative care [5], the term “palliative and hospice care” is used in this study as an umbrella term encompassing the full spectrum of holistic care for people with life-limiting illnesses, including medical, nursing, psychosocial, spiritual, and supportive care, across different settings.

In Germany, palliative and hospice care is provided through a combination of outpatient and inpatient services. Inpatient hospices are independent, freestanding, and primarily nurse-led facilities [6]. The number of beds ranges from 8 to12, and the average length of stay is approximately 3–4 weeks. Most patients remain in the hospice until death [7]. The provision of medical care is mostly undertaken by general physicians with basic palliative care knowledge or by physicians from an outpatient specialized palliative care team. Palliative care units are distinct from inpatient hospices and are integrated into hospitals as separate units. These units treat patients with particularly complex symptoms requiring continuous medical care. The goal of care is to control symptoms and facilitate discharge home or to other care facilities. The mean length of stay is 11.0 days [8]; over 60% of patients die during their stay on the palliative care unit. If discharge is possible, outpatient specialized palliative care is often involved due to their complex palliative care needs.

In this study, the term “academic” is defined according to the literature on academic health care. It refers to health care institutions that are integrated into a university and combine clinical care, research, and teaching within a single organizational structure [9,10]. Such settings link patient care directly to scientific inquiry and the education of healthcare professionals and play a key role in the translation and adoption of research findings into routine practice. Based on this understanding of academic health care, academic medical centers serve as best-practice models for the integration of innovations in order to provide high-quality care [11]. They also play a crucial role in palliative care by being involved in the training of healthcare professionals, conducting research to advance palliative care practices, and providing specialized clinical services [12–14]. Establishing inpatient hospices integrated into an academic medical center may offer several advantages, including enhanced interdisciplinary collaboration, access to specialized medical and nursing expertise, and expanded opportunities for education and research [15].

Both hospices and hospitals share historical roots in ancient Greek and Roman medicine [15]. The modern hospice movement, despite Cicely Saunders’ emphasis on evidence-based care [16], has largely developed from grassroots and community-driven initiatives. As a result, academic inpatient hospices remain uncommon, both in Germany and internationally. Existing examples of such integration is the inpatient hospice at University Medicine Greifswald, Germany [17], as well as St Gemma’s Hospice in Leeds, UK, which operates as a “University Teaching Hospice” in partnership with the University of Leeds [18]. To the best of our knowledge, they have not been systematically developed, implemented, or evaluated so far.

To inform the planning and design of an inpatient hospice in an academic setting, a preliminary interview study and survey were conducted with key stakeholders, including physicians, nurses, and hospice volunteers [19]. To sustainably enhance person-centered care, it is crucial to incorporate the needs and expectations of patients and their relatives into the planning and design of a new inpatient hospice [20]. This study explores the experiences, perceptions, and expectations of patients and their relatives regarding palliative and hospice care to inform the development of an inpatient hospice in an academic setting.

## Materials and methods

This study is reported following the Consolidated Criteria for Reporting Qualitative Research (COREQ) guidelines [21], (see S1 File). It is approved by the local ethics committee (reference No 23–1257), registered at the German Clinical Trials Register (#DRKS00034122), and conducted in accordance with the declaration of Helsinki [22].

### Design

This study employed a qualitative descriptive design with an exploratory focus to capture the subjective experiences, perceptions, and expectations of patients and their relatives regarding palliative and hospice care [23,24]. Semi-structured, guided interviews were conducted within a single-center setting to explore participants’ perspectives in depth. This methodological approach ensured close alignment with participants’ viewpoints generated practice-oriented findings that can directly inform the planning and development of an inpatient hospice within an academic context.

### Setting

This study was conducted at the Department of Palliative Medicine at the University Hospital of Cologne, Germany. The department provides a range of academic palliative and hospice care services across multiple sectors, including:

(1) Inpatient care: specialized 15-bed palliative care unit;
(2) Outpatient care: specialized palliative home care team providing care at patients’ homes, in long-term care facilities, and inpatient hospices;
(3) Consultative services: hospital inpatient palliative care support team offers consultation-based support across various non-palliative care departments;
(4) Volunteer-based services: hospice service offers support through professionally coordinated and trained volunteers;
(5) Palliative day care clinic: specialized daytime palliative care service providing medical assessment, symptom control, and therapy optimization without overnight admission.

The city of Cologne has a population of one million. There are four inpatient hospices run by different Christian organizations. Currently, the University Hospital of Cologne refers patients to these inpatient hospices, it as it does not operate its own facility.

### Participants

This study included patients with incurable, progressive diseases as well as their relatives who had direct or indirect experience with palliative and hospice care services in an academic setting. Relatives were defined as individuals involved in the patient’s care or of special importance to the patient. Eligible participants had to be over 18 years of age, fluent in German, and be able to give informed consent. Physicians and nurses of the Department of Palliative Medicine supported the recruitment process. They were informed about the study by the research team, provided with an information sheet, and asked to suggest eligible participants. Individuals who expressed prior interest in participation were subsequently contacted by the first author, either in person or by telephone. All potential participants received both oral and written information about the study. A purposive sampling strategy was employed to ensure diversity with respect to diagnosis, gender, age, and prior experience with palliative care, thereby capturing a broad range of perspectives. Based on existing literature on data saturation, the target sample size was set at 10–20 interviews [25]. Recruitment took place between April 17, 2024, and September 19, 2024.

### Data collection

An interview guide was developed by the research team based on the objectives of the study. The interview guide (see S2 File) included questions on the following topics:

(1) Personal and indirect experiences with palliative and hospice care
(2) Understanding of palliative and hospice care
(3) Perceptions of palliative and hospice care.
(4) Expectations of palliative and hospice care
(5) Recommendations for improving palliative and hospice care
(6) Attitudes and suggestions regarding an inpatient hospice in an academic setting

Participants were encouraged to talk about positive and negative experiences of palliative and hospice care. In-depth questions were used to elicit more specific information. Demographic data of participants were collected before the interviews and field notes were taken. The interviews were conducted either individually or jointly with patients and relatives, according to the participants preferences. The interviews were carried out in person or, if requested, by telephone. All interviews were conducted by the first author (ML; female, nurse; health services researcher (M.Sc.), trained and experienced in conducting interviews and not involved in the care). The audio recordings of the interviews were transcribed verbatim. The first author reviewed the transcripts and pseudonymized them prior to analysis to protect sensitive information. Transcripts were not returned to participants for comment or correction.

To ensure that quotations were accurately translated, they were first translated into English and then back into German using the translation software DeepL SE [26]. The transcripts were numbered and labeled according to whether the participant was a patient or a relative, as well as the palliative and hospice care service they received.

### Data analysis

**Fig 1:**
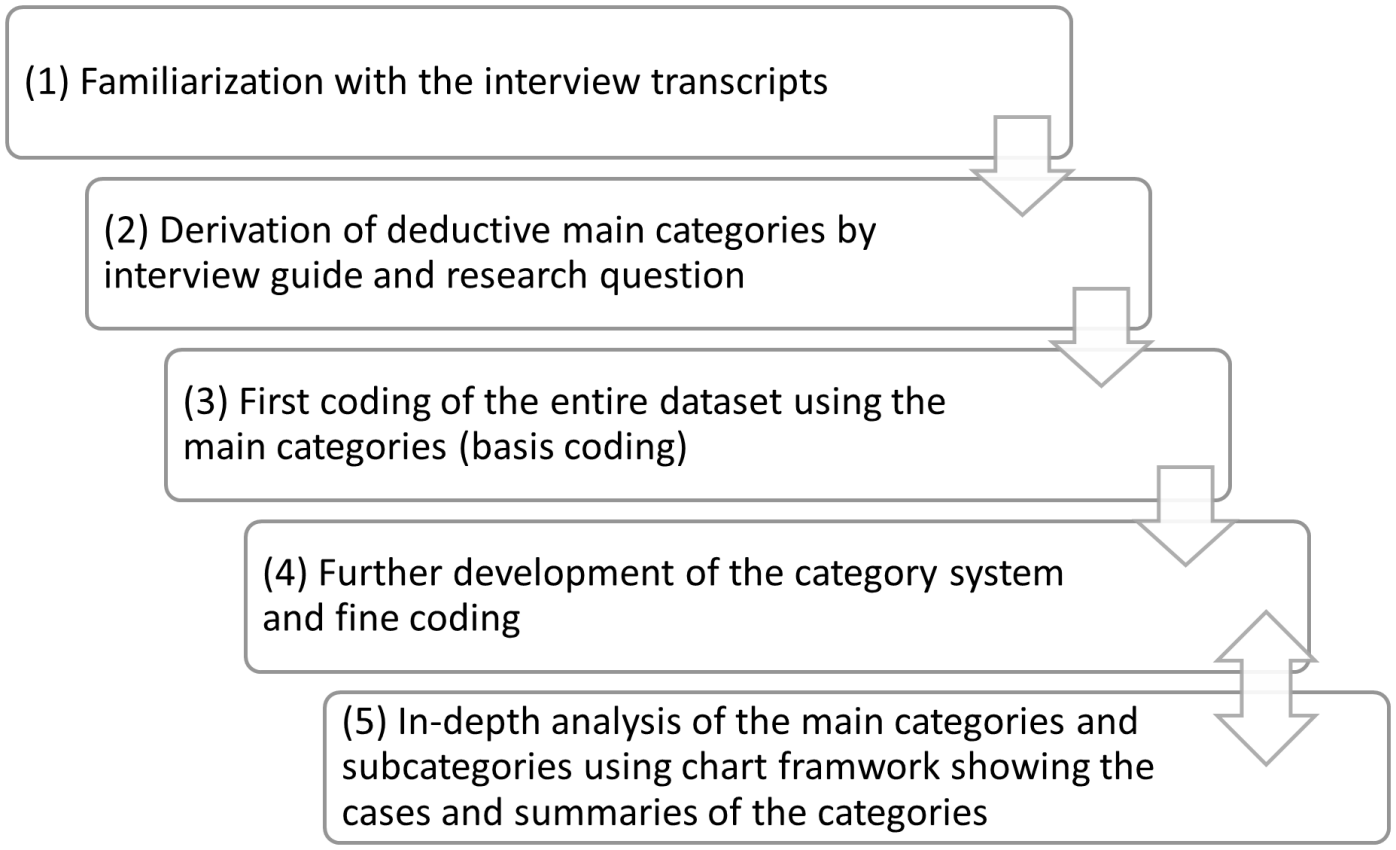
Steps of qualitative content analysis [27].

The data were analyzed using qualitative content analysis [27]. This process consists of five steps (Fig 1). Steps four and five involved a reflexive process that allows for shifts back and forth while ensuring that results remain rooted in the original data and their context.

The first author carried out the analysis steps, holding frequent meetings with KK (a senior researcher (PhD); experienced in qualitative research) to discuss the interim results of each step. The fine coding was independently verified by a second person (AW, research assistant, psychology student). Analysis was performed using the software MAXQDA 2024 [28]. The results of the content analysis were interpreted and discussed in the research team.

## Results

A total of 26 potential interview participants were identified by clinicians and members of the care teams, based on predefined inclusion and exclusion criteria. Of these, nine individuals could not be enrolled, either because contact could not be established, the patient had died before contact was made, or no response was received. Between May and September 2024, 14 interviews were conducted with a total of 17 participants, including ten patients and seven relatives. Interview duration ranged from 11 to 46 minutes. Eight individual interviews were conducted with patients and four with relatives. In addition, two dyadic interviews were carried out, in which patients and their relatives were interviewed together. Most interviews were conducted face-to-face: seven took place in the hospital, four in an inpatient hospice, and two in participants homes. One interview was conducted by telephone. All participants had experience with at least one form of palliative or hospice care services. Specifically, four participants had experience with specialized outpatient palliative care in an inpatient hospice setting, seven with specialized outpatient palliative care at home, three with hospital-based palliative care consultation teams, ten with palliative care units, and five with volunteer-based visiting services. The patients ranged in age from 45 and 92 years. The majority had been diagnosed with cancer (n = 12), with a disease duration ranging from one month to six years. Detailed characteristics of the interviewees and their experiences with palliative and hospice care services are presented in the case-based overview (see Table 1).

**Table 1:** Case-based overview of the characteristics and experiences of the interviewees with academic palliative and hospice services.

	Characteristics						Experience with academic palliative and hospice care services				
Case	Participant	Age of Patient	Gender of Patient	Disease of Patient	Duration of Disease	Relationship to Relative	Specialized outpatient palliative care in the hospice	Specialized outpatient palliative care at home	Palliative care unit	Hospital palliative care support team	Volunteer-based visitor service
**1**	Patient	92	Male	Cancer	1 Year	N/A	✓*	/	/	/	/
**2**	Patient	92	Female	Cancer	1 Year	N/A	/	✓*	/	/	✓
**3**	Relative	/	/	Cancer	/	Daughter	/	✓*	/	/	✓
**4**	Patient	82	Female	Cancer	2 Years	N/A	✓*	/	✓	/	/
**5**	Patient	79	Male	Cancer	2,5 Months	N/A	✓*	/	✓	/	/
**6**	Patient	46	Female	Cancer	6 Years	N/A	/	✓	/	✓*	/
**7**	Relative	/	/	Non-Cancer	/	Spouse	/	✓*	/	/	/
**8**	Patient; Relative 1; Relative 2	87	Male	Non-Cancer	/	Daughter Son	/	/	✓*	/	/
**9**	Relative	/	/	Cancer	/	Daughter	/	✓	✓*	/	/
**10**	Patient; Relative	62	Male	Cancer	4,5 Years	Spouse	/	✓	✓*	/	✓
**11**	Patient	45	Female	Cancer	1 Months	N/A	/	✓	/	✓*	✓
**12**	Relative	/	/	Cancer	/	Stepfather	/	/	✓*	/	/
**13**	Patient	50	Female	Cancer	4 Months	N/A	/	/	/	✓*	/
**14**	Patient	79	Male	Cancer	7 Months	N/A	✓	/	✓*	/	/

*Type of Service Used at Time of Recruitment.

Qualitative data analysis revealed three main categories: 1. Perception and attitudes toward palliative and hospice care, 2. Experiences with palliative and hospice care and 3. Wishes and expectations for palliative and hospice care. Table 2 shows these main categories and their sub-categories. Below, they are presented narratively and supported by quotes.

**Table 2:** Main and sub-categories resulted from qualitative data analysis [24].

Main-categories	Sub-categories	Description
1. Perception and attitudes toward palliative and hospice care	1.1 Uncertainties 1.2 Relieve suffering 1.3 Person-centered 1.4 End-of -Life 1.5 Change in attitude 1.6 Attitude toward an academic inpatient hospice	This category covers both aspects of the understanding of palliative and hospice care as well as the perception and change of attitudes.
2. Experience of palliative and hospice care	2.1 Psycho-social aspects of care 2.2 Physical aspects of care 2.3 Transitions, communication, and coordination of care 2.4 Setting and services offered in care	This category covers the experiences made in palliative and hospice care. The experiences are embedded in experiences in other palliative and hospice care settings and in curative care facilities.
3. Wishes and expectations for palliative and hospice care	3.1 Psycho-social aspects of care 3.2 Physical aspects of care 3.3. Transitions, communication, and coordination of care 3.4 Setting and services offered in care	This category covers what specific needs and concerns the participants have regarding these forms of care and what characteristics they would like to see in palliative and hospice care.

### Perception and attitudes of palliative and hospice care

The following sections describe participants perceptions and attitudes. They reflect participants understanding, values, and expectations regarding palliative and hospice care.

#### Uncertainties.

Participants frequently expressed uncertainty regarding the scope and definition of palliative and hospice care. In particular, they questioned whether and how the two forms of care differ, and which specific services each entails. These uncertainties appeared to influence their initial expectations and perceptions.

*“Is there a difference between palliative and hospice care? I thought that were the same thing.”* (Case 2, Patient)

#### Relieve suffering.

The relief of suffering, particularly through effective pain management, was consistently identified as a key aspect of palliative and hospice care. Participants emphasized that effective symptom control is a core element of their understanding of these services.

*“[…] you are in a lot of pain and are given pain medication so that you can bear it to some extent.”* (Case 10, Patient)

#### Person-centered.

Participants described palliative and hospice care as empathetic and holistic approaches, that address the person as a whole, rather than focusing solely on the underlying illness. Particular emphasis was placed on the recognition of individual needs, values, and life circumstances, which were perceived as defining characteristics of this model of care.

*“I assume that the focus is more on the whole person, including their medical history, which can also inevitably lead to death, which I believe is part of palliative care or a hospice.”* (Case 6, Patient)

#### End-of-life.

Participants viewed the inpatient hospice as the “last station” before death, whereas admission to a palliative care unit was perceived as an intermediate phase. This distinction shaped their understanding of the respective roles and purposes of these care settings. While the hospice was primarily associated with end-of-life care, the palliative care unit was viewed as part of a broader continuum of care, potentially including stabilization and subsequent discharge.

*“The inpatient hospice is a place where you just go in […] and that’s where you die.”*(Case 8, Relative)

*“Here [academic palliative care unit] that’s still a kind of intermediate space for me. You can also die here, but it’s not mainly specialized.”* (Case 10, Patient)

#### Change in attitude.

Several participants described that their initial skepticism toward palliative and hospice care changed following direct experience with these services. This change in attitude was associated with direct encounters with care staff and the care environment, which appeared to reshape participants perceptions of palliative and hospice care.

*“I was so pleasantly surprised, because somehow you always had it in your head to equalize it [note: the inpatient hospice] with a hospital and it’s not like that. It was cozy, it was familiar quite quickly, it was loving, it was empathetic, it was simply / it was great.”* (Case 9, Relative)

*“[...] Palliative, first of all fear. [...] I find it a bit negative and associated with fear. An uncomfortable feeling. […] and right now, in the current phase as I am here [note: academic palliative care unit], I really see it differently again and could also add to what I said earlier, a lot is done here to make you feel self-determined.”* (Case 10, Patient)

#### Attitudes toward an academic inpatient hospice.

Attitudes toward an inpatient hospice affiliated with an academic medical center were predominantly positive. Some participants had not yet formed an opinion, as they lacked a clear understanding of what such a facility would entail. Others expressed the expectation that it could expand the availability of hospice beds and enhance continuity of care during the transition from palliative to hospice services.

*“I think an expansion in this area can only ever be a benefit. I would wish every person who is ill that they had the opportunity to have a place like this, I can only support this.”* (Case 9, Relative)

Several participants highlighted the perceived advantages of integrating palliative and hospice care within a single facility and emphasized that a close link between the two types of care could be beneficial.

*“Why should you separate them? [...] I think it would even be an advantage if it was combined a bit.”* (Case 5, Patient)

Participants suggested that the familiar environment of an academic inpatient hospice could ease the transition for both patients and relatives by drawing on their prior experiences within the academic medical center. This familiarity was perceived as facilitating adaptation to the hospice setting and enhancing comfort.

*“It’s also a place that people are already used to, which can help many people to settle in.”* (Case 13, Patient)

Given the scope of the medical faculties, participants considered a hospice a logical addition to an academic medical center, offering multiple opportunities for enhanced care and integration of services.

*“The university hospital is a large facility here with many medical faculties and so many possibilities, I think that’s just part of it, so it’s so, so important.”* (Case 12, Relative)

### Experiences, wishes and expectations with regard to palliative and hospice care

The results for the main categories ‘Experiences with palliative and hospice care’ and ‘Wishes and expectations for palliative and hospice care’ are presented together to highlight the connections between previous care experiences and participants wishes and expectations concerning palliative and hospice care. The presentation is structured around four subcategories: (1) psycho-social aspects of care, (2) physical aspects of care, (3) transitions, communication, and coordination of care, and (4) setting and services offered in care. Within each subcategory, experiences are described first, followed by the corresponding wishes or expectations.

#### Psycho-social aspects of care.

Patients and relatives described emotional support in palliative and hospice care as providing a sense of security and relief, reducing feelings of isolation. Personal attention and the presence of staff and volunteers who offered comfort and companionship were emphasized.

*“And the human companionship - in other words, that you felt you were in good hands when you had fears and worries.”* (Case 7, Relative)

*“I felt so incredibly well understood here and [...] I felt deeply that it was all about me and making the last time somehow nice for me*. *”* (Case 10, Patient)

Participants valued the lively and positive atmosphere, noting that humor and friendly interactions contributed significantly to their comfort.

*“They’re funny, the people here have fun [...] it’s really great, I feel so comfortable here [in the hospice].”* (Case 4, Patient)

*“And here [academic palliative care unit] people in this condition are simply given a laugh.”* (Case 8, Patient)

Participants also reported that palliative and hospice care places a strong emphasis on preserving patients´ autonomy and dignity.

*“And here [in the hospice] I am [...] my free person, so [...] here you are still somehow a human being*.*”* (Case 4, Patient)

*“Here [on the academic palliative care unit], a lot is done to make you feel self-determined.”* (Case 10, Patient)

Participants highlighted that palliative and hospice care extends support beyond the individual to include relatives and the broader social environment. They described experiences in which the family and social network were acknowledged and supported, and they emphasized the expectation that care should continue to address relatives´ needs, ensuring they are neither overlooked nor left without guidance.

*“The entire system is acknowledged – the family, friendship network, our social environment, that’s how I would describe it, and it’s simply amazing.”* (Case 10, Relative)

*“It’s important to me that the family members don’t somehow get left behind. That they are well taken care of, so that they don’t fall into a hole, because they are quickly forgotten.”* (Case 10, Patient)

Some participants expressed a desire for more meaningful personal connections, particularly aligned with personal interests, with both professionals and volunteers in palliative and hospice care.

*“[…] so there should be a bit of chemistry, I mean there should be a wavelength that you can relate to together.”* (Case 11, Patient)

This extended to their desire for spiritual guidance that was not tied to a specific religion but rather reflected an overarching spirituality, one that acknowledged and respected personal choices, including the rejection of curative treatments.

*“I decided not to undergo radiotherapy [...] and if I were to die and hear something like that, I would find it terrible- ‘You could have had the radiotherapy.”* (Case 6, Patient)

It is expected that efforts are undertaken in palliative and hospice care to awaken people’s curiosity and support engagement in meaningful activities, and help maintain involvement in their daily life and interactions within their environment.

*“[…] how can I awaken the curiosity of old people to participate in life again [...]. And that is the main task of palliative care”* (Case 1, Patient)

#### Physical aspects of care.

Experiences of the physical aspects of care are characterized by symptom management, with pain management being the most frequently mentioned. Beyond pain, other symptoms such as nausea, diarrhea, and swollen legs were also treated. Participants described that effective symptom control contributed significantly to their comfort and well-being. One participant described that nutrition difficulties were managed positively with artificial feeding at night and light meals during the day.

*“Due to my illness, I also have extreme difficulties with food intake, and they handled it extremely positively.”* (Case 14, Patient)

Complementary therapies like aromatherapy and music therapy were valued for their sensory stimulation.

*“It’s good when you can pause and just smell or feel the water running over your arm. That’s nice. ”* (Case 10, Patient)

*“[…] you really felt how it went into the body. Dreamlike.”* (Case 1, Patient)

Another participant reported that stressful procedures such as blood tests or injections decreased.

*“[...] then I went from there [note: general inpatient unit] to the palliative care unit, I didn’t get any more injections, nothing more.”* (Case 5, Patient)

Beyond their experiences, participants expressed expectations for comprehensive physical care that goes beyond the immediate palliative diagnosis by addressing other health issues and side effects of their conditions.

*“They should maybe focus more on the health of the patient, not just the illness. Things like swollen feet, difficulty seeing, or other issues could be addressed more.”* (Case 4, Patient)

Participants highlighted mobility assistance as an important need, emphasizing the value of regular support for physical activities, such as walking, to maintain independence and preserve functional ability.

#### Transitions, communication and coordination in care.

Participants described their journey through the healthcare system, from initial hospitalizations with curative treatments to the transfer to palliative and hospice care, often contrasting the rushed hospital interactions with experiencing more personal and respectful communication in palliative and hospice settings, where care providers took time to engage with both patients and relatives.

*“You didn’t get the feeling that they were beating around the bush,[…] everything was discussed down to the smallest detail.”* (Case 9, Relative)

*“They lower themselves to the patient’s eye level, and I find that so important because then the person is truly seen.”* (Case 6, Patient)

The participants described their experiences with a calm environment, sufficient time devoted to each patient, and a consistently caring atmosphere in palliative and hospice care.

*“So here you somehow have people with an incredible amount of empathy and time who are available to you and so the approach [...]not somehow ‘now quickly the nursing care and then the next patient [...] so it’s not a functional mode.” (Case 10, Patient)*

The hospital inpatient palliative care support team provided both guidance and support, initiating specialized outpatient palliative care during the stay on the general unit. This approach ensured a coordinated transition from the hospital to the home environment.

*“I experienced support, exactly. That is also what I mean by support, and that is also a concrete offer, that you are not left alone when you leave hospital”* (Case 6, Patient)

The transition from an academic palliative care unit to an inpatient hospice was experienced initially with negative emotions. One participant (Case 4, Patient) felt like being *‘shunted off*’, though it was also reported that comfort was found in the inpatient hospice after the initial reluctance.

*”I was totally happy until they told me that I had to leave [the palliative care unit]. […] the doctor told me ‘it’s not possible’ and that I must go somewhere else, to be transferred to an inpatient hospice. I ended up here [in an inpatient hospice]. In principle, of necessity.”* (Case 5, Patient)

*“I think this is very difficult for the family, especially for the mother, because she is, first of all, very exhausted, of course [...] and doesn’t want to deal with a move”* (Case 12, Relative)

The need for clear and timely communication, particularly concerning the transition to inpatient hospice, was strongly emphasized. Additionally, some participants highlighted the importance of improved integration between palliative and hospice care services across both inpatient and outpatient settings.

*“I think it would have been better if there had been more contact with the hospice directly. We only had contact with the palliative team, not with the inpatient hospice itself.”* (Case 3, Relative)

#### Setting and service offered in care.

Participants with experience in inpatient hospices emphasized the homely atmosphere, noting features such as personalized rooms and amenities including laundry services, balconies and access to outdoor spaces. Both the academic palliative care unit and inpatient hospice were clearly distinguished from hospitals, being described as less institutional and more homelike, with single rooms. The option of moving the bed into the garden was particularly appreciated.

*“Then I was [...] lucky enough to have a room on the ground floor with a terrace outside. That was of course wonderful.”* (Case 14, Patient)

*“But in principle, if you’re used to a double room in hospital, then this [note: on the palliative care unit] is la dolce vita [...].”* (Case 10, Relative)

*“There’s nothing clinical about the whole atmosphere. Nobody walks around here in a white coat, there are no hierarchical rounds […].”* (Case 1, Patient)

Therapeutic offerings like art, music, physiotherapy, animal-assisted therapy and conversations with volunteer staff were highly valued.

*“ [...] and that’s why I think it’s very positive that there are volunteers who just have conversations with people like my mother.”* (Case 3, Relative)

*“When we talk about the hospice service, is a lady who has been assigned to me, who can do things with me, help me with errands, chat with me, and so on.”* (Case 13, Patient)

Participants expressed a desire for a holistic approach that includes alternative therapies, which they felt could contribute to overall well-being beyond medical treatment alone.

*“Alternative options [such as acupuncture]* that *I would of course wish for in such a case, simply because they contribute to the overall well-being of the person. This means not just medically, but holistically.”* (Case 13, Patient)

Some participants suggested creating a *‘community center’* or dedicated space for creative activities where patients and families could engage in shared activities or enjoy social interaction. Several participants also emphasized the need for quiet areas and spaces for reflection. Environmental improvements were recommended, including wooden interiors and larger, more accessible bathrooms. Concerns were raised regarding the limited menu options on the academic palliative care unit, particularly for patients requiring soft food, with suggestions to offer a greater variety. The importance of food was emphasized with one participant (Case 1, Patient) describing it as *‘one of the fundamental forms of human life’* underscoring its relevance for palliative and hospice care. Practical considerations such as transport and accessibility were also noted, alongside a preference for smaller, more personal care settings.

*“Still, I had this feeling like, there’s a hospice, there’s a palliative care unit, there’s a day clinic, and I wondered, how big will it be? Will it be the kind of building where you stand in front of it and think, ‘Oh my God ’ or something like that?”* (Case 10, Relative)

Technical and digital innovations were suggested to allow staff to spend more time in direct interaction with patients.

*“Why don’t we have a little robot running around here to clean and maybe train these people who are cleaning here now to do a better or different task that requires more of the human being [...] that leads back to the human.”* (Case 1, Patient)

Participants also suggested promoting the use of technological communication aids to facilitate social participation for individuals in palliative and hospice care*.* Additionally, concerns were raised regarding the funding of palliative and hospice services, particularly in the context of limited resources.

## Discussion

The aim of the study was to explore patients and relatives experiences, perceptions, and expectations regarding palliative and hospice care with the goal of informing the development of an inpatient hospice within an academic setting. Our findings provide a comprehensive, empirically grounded understanding of these perspectives and highlight which aspects patients and relatives consider particularly important for the development of an academic inpatient hospice.

### Experiences and perspectives on palliative and hospice care

Our findings indicate that patients and relatives perceive palliative and hospice care as a differentiated and multidimensional provision of care. Participants highlighted the emotional and social support they received from professionals and volunteers, emphasizing the importance of trusting, personal communication and moments of human closeness. Within these interactions, the preservation of autonomy and dignity was described as particularly meaningful. These psycho-social aspects were also highlighted in other studies [29–32], confirming that such support is a recognized core component of high-quality hospice and-palliative care.

Symptom management was consistently described as a core strength of care, with participants valuing effective pain relief and attention to other burdensome symptoms. Beyond symptom control, participants appreciated attention to additional health issues and efforts to provide a holistic approach, which aligns with prior studies emphasizing comprehensive, person-centered care [33,34].

A ‘homely’ atmosphere in care settings, personalized room design, access to outdoor spaces, including movable beds to a garden area and shared space for social interaction were highly valued. Other studies have also explored architecture-related aspects in palliative and hospice care facilities and confirm our findings in this regard [35–38].

Taken together, these findings underline that palliative and hospice care already fulfill essential functions in supporting seriously ill persons and their relatives as also emphasized in a recent study by Hughes et al. [39]. At the same time, it is crucial to preserve and strengthen palliative and hospice care while further advancing and expanding them to better meet the needs and preferences of patients and their relatives – especially in light of demographic developments, financial constraints and resource limitations.

### Potential contributions of an academic inpatient hospice

Based on the perspectives of patients and relatives in this study, several areas emerged where an academic inpatient hospice could provide valuable support and improvements. Among these, uncertainties and misconceptions about palliative and hospice care were frequently mentioned. Such uncertainties may contribute to negative perceptions and hesitant attitudes, which participants often hold prior to gaining firsthand experience, a phenomenon also observed in other studies [29,40–42]. Misconceptions about palliative and hospice care are widespread in the general population, with many people having a vague understanding of goals and responsibilities of this care [43–45]. An academic inpatient hospice could contribute to raising society’s awareness of palliative and hospice care by integrating education and research activities alongside patient care. Educational programs can primarily target healthcare professionals, volunteers, but also include public lectures. By fulfilling the university’s societal mission (“Third Mission”), these programs help sensitize the public to relevant social topics and promote understanding.

Participants articulated a need for emotional and spiritual support, independent of a particular religion, as well as for continuous accompaniment following the discontinuation of curative treatment. In our study, most participants were patients with cancer, but the need for holistic, person-centered care extends to patients with other serious illnesses, who may have different trajectories and often access palliative services later or less frequently [46]. An academic inpatient hospice could address these needs by providing care that integrates physical, emotional, social, and spiritual support. By beeing closely linked to an ancademic medical center, it can also demonstrate that opitmal care of the dying is an integral part of comprehensive, high-quality healthcare. Embedding a hospice within such an academic medical center could help ensure that holistic, person-centered care is considered [47].

The transition from a hospital-based palliative care unit to a freestanding inpatient hospice was described as emotionally distressing. Previous research has similarly identified end-of-life transitions as emotionally challenging, often marked by insufficient coordination, unclear expectations, and a lack of relational continuity [48–50]. These findings can be interpreted through the concept of continuity of care, which includes informational, management, and relational dimensions [51]. Transitions should ensure that relevant information is carried forward, care adapts consistently to changing needs, and established relationships with all those involved in care are preserved. An academic inpatient hospice can reduce the need for external placements by coordinating transitions and enabling internal transfers. Bringing palliative and hospice care together under one roof minimizes disruptions and strengthen informational, management, and relational continuity.

Participants explicitly expressed a desire for architectural innovations, emphasizing the need for structural improvements that create a supportive, flexible, and accessible environment tailored to the needs of patients and their relatives. This includes designing spaces that foster a homely atmosphere, facilitate privacy, and enable social interaction. In addition, participants highlighted the importance of integrating digital and technical solutions. Such design considerations align with evidence-based design, which emphasizes the use of empirical research to create healthcare environments that promote patient comfort, safety and the well-being of staff [52]. An academic inpatient hospice could apply evidence-based design principles to create environments that promote comfort and well-being. Additionally, technical and digital innovations could be developed, tested and integrated.

Overall, attitudes toward an academic inpatient hospice were predominantly positive. Participants emphasized the hope for an expansion of inpatient hospice places and appreciated the close connection between palliative and hospice care. They also highlighted the potential of integrating an inpatient hospice within an academic medical center to support high-quality care. Finally, although participants did not explicitly address research activities, their expressed desire for individualized care, continuity, and ongoing improvement points to the potential value of integrating systematic research into clinical practice. Embedding research within an academic inpatient hospice could help evaluate and refine interventions, in line with the broader role of academic health science systems in linking care, research, and education [15].

### Strengths and limitations

A strength of the study lies in the qualitative approach, which provides deeper insights into the experiences, perceptions and expectations of patients and relatives. This is the first study to relate their perspectives on an academic inpatient hospice, offering suggestions for its development. Although this study focuses on the development of an academic inpatient hospice in Germany, the findings have broader international relevance. By systematically capturing the experiences and expectations of patients and relatives, the study highlights which aspects of care, support, and environment are most valued. These insights can inform the design and implementation of patient-centered palliative and hospice care models worldwide, contributing to the global discourse on compassionate, relational, and meaningful end-of-life care [53,54].

A limitation is that most participants found it difficult to imagine what an academic inpatient hospice would entail. As described elsewhere, patients and relatives often express general value expectations rather than specific (predictive) expectations toward unfamiliar models of care [55]. In addition, the different definitions and understandings of palliative and hospice care could have led to differences in interpretation in the participants descriptions and explanations. This should be taken into account when interpreting the results. Furthermore, the study’s findings may not be fully transferable to other contexts or regions. Social desirability effects could also have meant that critical points were not addressed openly although this was reduced by the fact that the interviews were conducted by a person who does not work in direct care and created an atmosphere of trust. Additionally, the study mainly included cancer patients and future research should explore perspectives of non-oncological patients and those from different cultural and social backgrounds in order to gain more comprehensive understanding of the experiences and expectations of care. Further research could also examine which specific interactions, care aspects, or patient characteristics shape patients and relatives perspectives on an academic inpatient hospice.

## Conclusion

This study provides valuable insights into the expectations and needs of patients and relatives with regard to palliative and hospice care, supporting the development of an academic inpatient hospice. While previous research has already explored the perspectives of healthcare professionals and key stakeholders [19], further investigation is required to clarify the structural, legal, and organizational frameworks essential for implementation. Moreover, it is important to further elaborate on how academic inpatient hospices can effectively support and strengthen palliative and hospice care. Key domains can be identified in which an academic inpatient hospice could make a meaningful contribution, reflecting both the needs and expectations of patients and relatives as well as broader societal, structural, and research-related considerations:

1. Education and Awareness – reducing misconceptions and increasing understanding among healthcare professionals, volunteers, and the public.
2. Holistic and Person-Centered Care – providing comprehensive support that addresses patients’ emotional, spiritual, and physical needs.
3. Transition and Continuity – ensuring seamless transitions between care settings, preserving informational, management, and relational continuity.
4. Architecture and Innovation – designing spaces that promote comfort, privacy, and social interaction, and integrating digital and technical solutions to enhance care and safety.
5. Integration of Research – embedding research and evaluation to inform practice, improve care quality, and support innovation in end-of-life care.

These domains illustrate the multiple ways an academic inpatient hospice can add value, providing comprehensive and responsive care that meets patient and societal needs while promoting innovation and evidence-informed practice.

## Supporting information

S1 File Consolidated criteria for reporting qualitative research (COREQ) guidelines. (DOCX)

S2 File Semi-structured initial interview guide. (DOCX)

## References

[1] WHPCA. Global Atlas of Palliative Care. London, UK. 2020. https://www.palliativecare.in/wp-content/uploads/2020/10/Global-Atlas-2nd-Edition-2020.pdf

[2] Scholten N, Günther AL, Pfaff H, Karbach U. The size of the population potentially in need of palliative care in Germany--an estimation based on death registration data. BMC Palliat Care. 2016;15:29. doi: 10.1186/s12904-016-0099-2 26957121 PMC4782573

[3] Etkind SN, Bone AE, Gomes B, Lovell N, Evans CJ, Higginson IJ, et al. How many people will need palliative care in 2040? Past trends, future projections and implications for services. BMC Med. 2017;15(1):102. doi: 10.1186/s12916-017-0860-2 28514961 PMC5436458

[4] Groeneveld EI, Cassel JB, Bausewein C, Csikós Á, Krajnik M, Ryan K, et al. Funding models in palliative care: Lessons from international experience. Palliat Med. 2017;31(4):296–305. doi: 10.1177/0269216316689015 28156188 PMC5405831

[5] Hui D, De La Cruz M, Mori M, Parsons HA, Kwon JH, Torres-Vigil I, et al. Concepts and definitions for “supportive care,” “best supportive care,” “palliative care,” and “hospice care” in the published literature, dictionaries, and textbooks. Support Care Cancer. 2013;21(3):659–85. doi: 10.1007/s00520-012-1564-y 22936493 PMC3781012

[6] German Guideline Program in Oncology. Extended S3 Guideline. Palliative care for patients with incurable cancer. 2020. https://www.leitlinienprogramm-onkologie.de/fileadmin/user_upload/Downloads/Leitlinien/Palliativmedizin/Version_2/GGPO_Palliative_Care_ShortVersion_2.2.pdf

[7] Kaiser U, Vehling-Kaiser U, Hoffmann A, Kaiser F. Inpatient hospices in Germany: medical care situation and use of supportive oncological therapies for symptom control in tumor patients. Palliative Medicine Reports. 2022;3(1):169–80. 36059908 10.1089/pmr.2022.0026 PMC9438444

[8] Dasch B, Melching H, Maier BO, Lenz P, Bausewein C, Rosenbruch J. A Nationwide Survey of Palliative Care Units in Germany on Structures and Patient Care. Dtsch Arztebl Int. 2024;121(3):92–3. doi: 10.3238/arztebl.m2023.0251 38471182 PMC11002437

[9] Dzau VJ, Ackerly DC, Sutton-Wallace P, Merson MH, Williams RS, Krishnan KR, et al. The role of academic health science systems in the transformation of medicine. Lancet. 2010;375(9718):949–53. doi: 10.1016/S0140-6736(09)61082-5 19800111

[10] Delaney B, Moxham J, Lechler R. Academic health sciences centres: an opportunity to improve services, teaching, and research. Br J Gen Pract. 2010;60(579):719–20. doi: 10.3399/bjgp10X532620 20883620 PMC2944930

[11] Ellner AL, Stout S, Sullivan EE, Griffiths EP, Mountjoy A, Phillips RS. Health Systems Innovation at Academic Health Centers: Leading in a New Era of Health Care Delivery. Acad Med. 2015;90(7):872–80. doi: 10.1097/ACM.0000000000000679 25738387

[12] Dronia M-C, Dillen K, Elsner F, Schallenburger M, Neukirchen M, Hagemeier A, et al. Palliative care education and knowledge transfer into practice - a multicenter survey among medical students and resident physicians in Germany using a mixed-methods design. GMS J Med Educ. 2024;41(3):Doc27. doi: 10.3205/zma001682 39131897 PMC11310786

[13] Paes P, Ellershaw J, Khodabukus A, O’Brien B. Palliative care in acute hospitals - a new vision. Future Healthc J. 2018;5(1):15–20. doi: 10.7861/futurehosp.5-1-15 31098525 PMC6510048

[14] Eichenauer DA, Golla H, Thielen I, Hallek M, Voltz R, Perrar KM. Characteristics and course of patients with advanced hematologic malignancies receiving specialized inpatient palliative care at a German university hospital. Ann Hematol. 2019;98(11):2605–7. doi: 10.1007/s00277-019-03748-1 31253997

[15] von Gunten CF, Ryndes T. The academic hospice. Ann Intern Med. 2005;143(9):655–8. doi: 10.7326/0003-4819-143-9-200511010-00009 16263888

[16] Saunders C. The evolution of palliative care. Patient Educ Couns. 2000;41(1):7–13. doi: 10.1016/s0738-3991(00)00110-5 10900362

[17] Universitätsmedizin Greifswald. Geschäftsbereiche des Pflegevorstands. Greifswalder Hospiz am Universitätsklinikum Greifswald. https://www.medizin.uni-greifswald.de/de/ueber-die-umg/geschaeftsbereiche-zentrale-bereiche/hospiz/

[18] St Gemma’s Academic Unit of Palliative Care. Academic Unit of Palliative Care Annual Report 2025. 2025. https://www.st-gemma.co.uk/app/uploads/2025/11/St-Gemmas-AUPC-Annual-Report-DIGITAL.pdf

[19] Dillen K, Montag T, Weihrauch B, Golla H, Voltz R, Strupp J. “Such an institution represents the circle of life” - bringing an inpatient hospice into an academic setting: a pre-implementation exploratory study. BMC Palliat Care. 2023;22(1):96. doi: 10.1186/s12904-023-01220-6 37464336 PMC10354892

[20] Rumbold B, Aoun SM. Palliative and End-of-Life Care Service Models: To What Extent Are Consumer Perspectives Considered?. Healthcare (Basel). 2021;9(10):1286. doi: 10.3390/healthcare9101286 34682966 PMC8536088

[21] Tong A, Sainsbury P, Craig J. Consolidated criteria for reporting qualitative research (COREQ): a 32-item checklist for interviews and focus groups. Int J Qual Health Care. 2007;19(6):349–57. doi: 10.1093/intqhc/mzm042 17872937

[22] General Assembly of the World Medical Association. World Medical Association Declaration of Helsinki: ethical principles for medical research involving human subjects. The Journal of the American College of Dentists. 2014;81(3):14–8. https://jamanetwork.com/journals/jama/fullarticle/10.1001/jama.2013.281053 25951678

[23] Bradshaw C, Atkinson S, Doody O. Employing a Qualitative Description Approach in Health Care Research. Glob Qual Nurs Res. 2017;4:2333393617742282. doi: 10.1177/2333393617742282 29204457 PMC5703087

[24] Kim H, Sefcik JS, Bradway C. Characteristics of Qualitative Descriptive Studies: A Systematic Review. Res Nurs Health. 2017;40(1):23–42. doi: 10.1002/nur.21768 27686751 PMC5225027

[25] Hennink M, Kaiser BN. Sample sizes for saturation in qualitative research: A systematic review of empirical tests. Soc Sci Med. 2022;292:114523. doi: 10.1016/j.socscimed.2021.114523 34785096

[26] Deepl SE. DeepL Translate. Cologne, Germany. 2025.

[27] Kuckartz U, Rädiker S. Qualitative content analysis: methods, practice and software. 55 City Road: SAGE Publications Ltd. 2023. 10.4135/9781036212940

[28] VERBI Software. MAXQDA 2024. Berlin, Germany: VERBI Software. 2023.

[29] Masel EK, Kitta A, Huber P, Rumpold T, Unseld M, Schur S, et al. What Makes a Good Palliative Care Physician? A Qualitative Study about the Patient’s Expectations and Needs when Being Admitted to a Palliative Care Unit. PLoS One. 2016;11(7):e0158830. doi: 10.1371/journal.pone.0158830 27389693 PMC4936709

[30] Michel C, Seipp H, Kuss K, Hach M, Kussin A, Riera-Knorrenschild J, et al. Key aspects of psychosocial needs in palliative care - a qualitative analysis within the setting of a palliative care unit in comparison with specialised palliative home care. BMC Palliat Care. 2023;22(1):100. doi: 10.1186/s12904-023-01227-z 37480117 PMC10360287

[31] Hughes NM, Noyes J, Eckley L, Pritchard T. What do patients and family-caregivers value from hospice care? A systematic mixed studies review. BMC Palliat Care. 2019;18(1):18. doi: 10.1186/s12904-019-0401-1 30736788 PMC6368799

[32] Bradley N, Dowrick C, Lloyd-Williams M. Explaining how and why social support groups in hospice day services benefit palliative care patients, for whom, and in what circumstances. Palliat Care Soc Pract. 2023;17:26323524231214549. doi: 10.1177/26323524231214549 38044931 PMC10693225

[33] Hannon B, Swami N, Rodin G, Pope A, Zimmermann C. Experiences of patients and caregivers with early palliative care: A qualitative study. Palliat Med. 2017;31(1):72–81. doi: 10.1177/0269216316649126 27495814

[34] Beernaert K, Deliens L, Vleminck A de, Devroey D, Pardon K, et al. Is there a need for early palliative care in patients with life-limiting illnesses? Interview study with patients about experienced care needs from diagnosis onward. The American Journal of Hospice & Palliative Care. 2016;33(5):489–97. doi: 10.1177/1049909115577352 25852203

[35] Beuls I, Petermans A, Vanrie J. Connect, care, create: practical framework for physical environment design for person-centred palliative care. BMJ Support Palliat Care. 2025;16(1):198–206. doi: 10.1136/spcare-2024-004805 39472028

[36] Sagha Zadeh R, Eshelman P, Setla J, Kennedy L, Hon E, Basara A. Environmental Design for End-of-Life Care: An Integrative Review on Improving the Quality of Life and Managing Symptoms for Patients in Institutional Settings. J Pain Symptom Manage. 2018;55(3):1018–34. doi: 10.1016/j.jpainsymman.2017.09.011 28935129 PMC5856462

[37] Abdel-Razek SA. Development of a user-centered design framework for palliative and hospice care patients for a better quality of life experience. ESSD. 2022;7(1):105–17. doi: 10.21625/essd.v7i1.870

[38] Annemans M, Coomans K, Heylighen A. Building scale and well-being in a hospice: a qualitative exploration. BMJ Support Palliat Care. 2022;12(e4):e505–9. doi: 10.1136/bmjspcare-2019-002151 32409570

[39] Hughes NM, Noyes J, Stringer C, Pritchard T. “Before I came to the hospice, I had nobody”. A qualitative exploration of what patients, family-caregivers, clinicians and volunteers valued most about home, day therapy or inpatient hospice services. Palliat Care Soc Pract. 2024;18:26323524241231820. doi: 10.1177/26323524241231820 38426037 PMC10903190

[40] El-Jawahri A, Traeger L, Shin JA, Knight H, Mirabeau-Beale K, Fishbein J, et al. Qualitative Study of Patients’ and Caregivers’ Perceptions and Information Preferences About Hospice. J Palliat Med. 2017;20(7):759–66. doi: 10.1089/jpm.2016.0104 28557586 PMC5695752

[41] Wallace CL, Coccia K, Khoo YM, Bullock K. Meaning of Hospice Care: Perceptions of Patients and Families. Am J Hosp Palliat Care. 2023;40(10):1132–40. doi: 10.1177/10499091221149702 36594567

[42] Tate CE, Venechuk G, Brereton EJ, Ingle P, Allen LA. It’s Like a Death Sentence but It Really Isn’t: What Patients and Families Want to Know About Hospice Care When Making End-of-Life Decisions. The American Journal of Hospice & Palliative Care. 2020;37(9):721–7. https://journals.sagepub.com/doi/10.1177/1049909119897259 31888342 10.1177/1049909119897259 PMC8403502

[43] Heckel M, Peters J, Schweighart S, Habermann M, Ostgathe C. Knowledge and Public Perception of Palliative Care in Germany. J Palliat Med. 2024;27(8):986–92. doi: 10.1089/jpm.2023.0630 38625024

[44] Batzler Y-N, Schallenburger M, Schwartz J, Marazia C, Neukirchen M. The General Public and Young Adults’ Knowledge and Perception of Palliative Care: A Systematic Review. Healthcare (Basel). 2024;12(10):957. doi: 10.3390/healthcare12100957 38786369 PMC11121430

[45] Taber JM, Ellis EM, Reblin M, Ellington L, Ferrer RA. Knowledge of and beliefs about palliative care in a nationally-representative U.S. sample. PLoS One. 2019;14(8):e0219074. doi: 10.1371/journal.pone.0219074 31415570 PMC6695129

[46] Kasdorf A, Dust G, Hamacher S, Schippel N, Rietz C. The last year of life for patients dying from cancer vs. non-cancer causes: a retrospective cross-sectional survey of bereaved relatives.Supportive Care in Cancer. 2022;30(6):4971–9. doi: 10.1007/s00520-022-06908-8 35190893 PMC9046331

[47] Janerka C, Leslie GD, Gill FJ. Development of patient-centred care in acute hospital settings: A meta-narrative review. Int J Nurs Stud. 2023;140:104465. doi: 10.1016/j.ijnurstu.2023.104465 36857979

[48] Hanratty B, Lowson E, Grande G, Payne S, Addington-Hall J, Valtorta N, Seymour J. Transitions at the end of life for older adults – patient, carer and professional perspectives: a mixed-methods study. Southampton (UK): NIHR Journals Library; 2014. 25642566

[49] Guo P, Pinto C, Edwards B, Pask S, Firth A, O’Brien S, et al. Experiences of transitioning between settings of care from the perspectives of patients with advanced illness receiving specialist palliative care and their family caregivers: A qualitative interview study. Palliat Med. 2022;36(1):124–34. doi: 10.1177/02692163211043371 34477022 PMC8793309

[50] Whitehead K, Ala-Leppilampi K, Lee B, Menagh J, Spaner D. Factors That Determine the Experience of Transition to an Inpatient Palliative Care Unit for Patients and Caregivers: A Qualitative Study. J Palliat Care. 2022;37(4):579–85. doi: 10.1177/08258597221105001 35837725 PMC9465532

[51] Haggerty JL, Reid RJ, Freeman GK, Starfield BH, Adair CE, McKendry R. Continuity of care: a multidisciplinary review. BMJ. 2003;327(7425):1219–21. doi: 10.1136/bmj.327.7425.1219 14630762 PMC274066

[52] Ulrich RS, Berry LL, Quan X, Parish JT. A conceptual framework for the domain of evidence-based design. HERD. 2010;4(1):95–114. doi: 10.1177/193758671000400107 21162431

[53] Sallnow L, Smith R, Ahmedzai SH, Bhadelia A, Chamberlain C, Cong Y, et al. Report of the Lancet Commission on the Value of Death: bringing death back into life. Lancet. 2022;399(10327):837–84. doi: 10.1016/S0140-6736(21)02314-X 35114146 PMC8803389

[54] Rodin G, Feldman A, Trapani D, Skelton M, Unger-Saldaña K, Essue B, et al. The human crisis in cancer: a Lancet Oncology Commission. Lancet Oncol. 2025;26(12):e628–70. doi: 10.1016/S1470-2045(25)00530-3 41192457

[55] van Klinken M, Graaf E de, Bressers R, Koorn R, van der Baan F. What do future hospice patients expect of hospice care: expectations of patients in the palliative phase who might be in need of hospice care in the future: a qualitative exploration. The American Journal of Hospice & Palliative Care. 2020;37(6):439–47. doi: 10.1177/1049909119893358 31818118 PMC7168802

