# Supplementary material for: Perspectives on hospice and palliative care in an academic setting: An exploratory interview study with patients and relatives to inform the development of an academic inpatient hospice
Source: PLoS One. 2026 May 7;21(5):e0348513. doi: 10.1371/journal.pone.0348513 (PMC13152117; doi:10.1371/journal.pone.0348513)
Supplement: S2 File — (DOCX) [file pone.0348513.s002.docx]

**Additional file 1: Semi-structured initial interview guide**

**Opening Question**

„You have been invited to take part in this interview because you have experience of hospice and palliative care. Today we would like to talk about your personal views on inpatient hospice and palliative care and find out what your wishes, expectations and experiences are in this regard.“

**Entry Questions**

„What specific experience have you gained in hospice and palliative care (direct/indirect)?”

- If inpatient experience is reported, follow up on this. If not, ask
- „What makes the care in an inpatient hospice special for you?“
- „What do you think palliative care is all about?“
- „In your opinion, what are the similarities/differences between a palliative care ward and a hospice?“

**Key Questions**

1. If you have experience of palliative care ward (direct/indirect):

- „ What did you find **positive** about the care on the palliative care ward?“
- „What did you find **negative** about the care on the palliative care ward?“
- „What do you **want** from care on a palliative care ward? Do you have any hopes for it, if so, what are they?”
- „Do you have any **fears or concerns** in this regard? If so, what are they?”
- „Do you see **potential for improvement**? If so, what? (For yourself, for your relatives, beyond that?)“

1. If there is experience with an inpatient hospice (direct/indirect):

- „What did you find **positive** about the care in the inpatient hospice?“
- „What did you find **negative** about the care you received in an inpatient hospice?”
- „What would you like to see from hospice care? Do you have any **hopes** for it, if so, what are they?”
- „Do you have any **fears or concerns** in this regard? If so, what are they?”
- „Do you see **potential for improvement**? If so, what? (For yourself, for your relatives, beyond that?)“

1. „We are planning the first university hospice and palliative care centre in Cologne, i.e. the various palliative and hospice structures are to be offered within one centre, including an inpatient hospice and a palliative care ward: what would you like to tell us for this planning? What suggestions do you have for us from your perspective? What is particularly important for you to pass on to us?”

**Final Question**

„We have already discussed a number of things. Is there anything else on this topic that has not yet been addressed but that you would like to say in conclusion?”
